# Ablação de Taquicardia Reentrante Nodal Atrioventricular com Crioablação Focal, Comparada com Ablação por Radiofrequência: Experiência em Centro Único

**DOI:** 10.36660/abc.20230604

**Published:** 2024-09-17

**Authors:** Caner Topaloğlu, Francesco Fici, Philippe van de Borne, Uğur Taşkin, Mustafa Dogdus, Serkan Saygi, Istemihan Tengiz

**Affiliations:** 1 Izmir University of Economics Department of Cardiology Izmir Turquia Izmir University of Economics - Department of Cardiology, Izmir – Turquia; 2 University of Salamanca Department of Cardiovascular Risk Salamanca Espanha University of Salamanca - Department of Cardiovascular Risk, Salamanca – Espanha; 3 University of Milan-Bicocca Milano Itália University of Milan-Bicocca – Cardiology, Milano – Itália; 4 Université Libre de Bruxelles Department of Cardiology Bruxelles Bélgica Université Libre de Bruxelles - Department of Cardiology, Bruxelles – Bélgica; 5 Erasmus Hospital Department of Cardiology Bruxelles Bélgica Erasmus Hospital - Department of Cardiology, Bruxelles – Bélgica; 6 Karolinska University Hospital Stockholm Suécia Karolinska University Hospital – Cardiology, Stockholm – Suécia

**Keywords:** Taquicardia por Reentrada no Nó Atrioventricular, Criocirurgia, Ablação por Radiofrequência

## Abstract

**Fundamento:**

A ablação da taquicardia por reentrada nodal atrioventricular (TRNAV) com crioablação é uma alternativa à ablação por radiofrequência (RF) em pacientes devido ao baixo risco de bloqueio atrioventricular total. Um aumento nas recorrências precoces e tardias após a crioablação é relatado como uma desvantagem importante.

**Objetivos:**

Neste estudo, objetivamos comparar o sucesso do procedimento agudo e as taxas de recorrência em longo prazo de pacientes com TRNAV submetidos a métodos.

**Métodos:**

Foram incluídos no estudo 73 pacientes com TRNAV: 32 com crioablação e 41 com ablação por RF. Não houve diferença estatisticamente significativa entre o sucesso agudo do procedimento nos métodos. O procedimento de ablação foi realizado por operador com experiência em arritmologia. A escolha entre RF ou crioablação foi feita no laboratório de eletrofisiologia com base no material já disponível durante o procedimento. Após o procedimento, os pacientes foram avaliados a cada 3 meses durante 2 anos em controle policlínico. O nível de significância adotado na análise estatística foi de 5%.

**Resultados:**

Os dois grupos de pacientes foram homogêneos. O tempo de fluoroscopia (p<0,001) foi menor, mas os tempos his-átrio (p=0,004) e his-ventricular (p=0,015) foram maiores no grupo crioablação. Não houve diferença significativa em termos de sucesso agudo do procedimento, salto pós-procedimento sem eco único e presença de eco e salto.

**Conclusões:**

A crioablação requer menos tempo de fluoroscopia e é uma alternativa segura e não inferior à ablação por RF em pacientes com TRNAV. O risco de bloqueio AV é um problema significativo com o uso de energia de RF, tornando-o menos adequado para uso em pacientes jovens e fisicamente ativos.

## Graphical abstract

**Figura Central:**
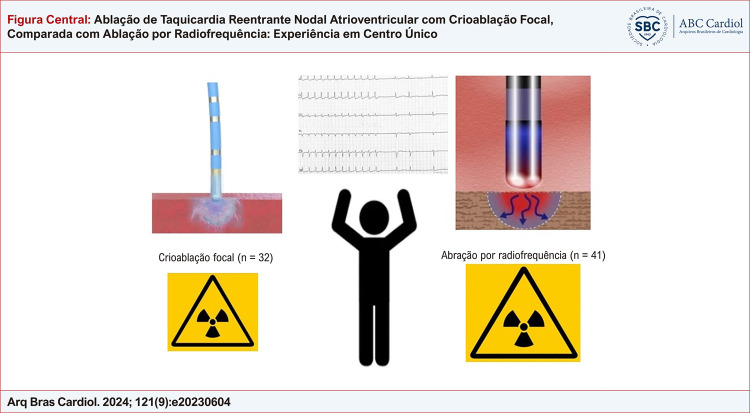
: Ablação de Taquicardia Reentrante Nodal Atrioventricular com Crioablação Focal, Comparada com Ablação por Radiofrequência: Experiência em Centro Único

## Introdução

Taquicardia supraventricular (TVS) é um termo geral para descrever um grupo de arritmias, cujo mecanismo inclui ou está acima do nó atrioventricular. A taquicardia por reentrada nodal atrioventricular (TRNAV) é a TVS mais comum em adultos, com prevalência global de 0,2%.^1-3^ Os mecanismos da grande maioria das formas de TVS foram elucidados e desenvolvimentos significativos foram alcançados nos tratamentos farmacológicos e intervencionistas.^4^

Geralmente, as diretrizes^5^ recomendam a ablação para pacientes com TVS recorrente ou mal tolerada, apesar do tratamento farmacológico. Quando as recomendações das diretrizes atuais são consideradas, a ablação por cateter é a modalidade de tratamento preferida para a maioria dos pacientes, independentemente da presença de doença cardíaca estrutural. Atualmente, existem duas opções para ablação por via lenta: crioablação focal e ablação por radiofrequência (RF). Ambas as técnicas têm diferentes vantagens e desvantagens.^6^

A crioablação focal é usada como alternativa à ablação por RF em pacientes selecionados devido à ausência de risco de bloqueio atrioventricular (AV) permanente. No entanto, um aumento nas recorrências precoces e tardias após a crioablação, em comparação com a ablação por RF, é relatado como a desvantagem mais importante deste método.^7^

## Objetivo

Nosso estudo retrospectivo teve como objetivo comparar a crioablação com a ablação por RF em termos de taxa de sucesso do procedimento e resultados de acompanhamento em longo prazo de pacientes com TRNAV.

## Métodos

O presente estudo retrospectivo foi realizado em pacientes submetidos à ablação da via lenta do nó AV para TRNAV. Setenta e três pacientes, selecionados a partir de prontuários do banco de dados do laboratório de cateterismo, foram incluídos no estudo. Toda a população do estudo foi composta por pacientes (73 pacientes) submetidos à ablação com diagnóstico de TRNAV nos 6 meses entre janeiro de 2020 e junho de 2020. Nenhum paciente foi excluído da análise. O procedimento de ablação foi realizado por um operador altamente experiente na área de arritmologia. A escolha entre RF ou crioablação foi feita no laboratório de eletrofisiologia com base no material já disponível durante o procedimento. Não houve outro viés em termos de escolha da técnica. O operador realizava rotineiramente os procedimentos de ablação todas as tardes dos dias úteis. O custo dos materiais utilizados em ambas as técnicas não foi oneroso para a sociedade e/ou para o paciente e não afetou a técnica escolhida. O estudo foi conduzido seguindo a Declaração de Helsinque e foi aprovado por um Comitê de Ética local independente.

A TRNAV foi diagnosticada usando os seguintes critérios, de acordo com relatos anteriores: (1) evidência de fisiologia nodal AV dupla, (2) início de taquicardia por trem de acionamento atrial com resposta Atrial-His-Atrial (A-H-A), (3) tempo septal ventrículo-atrial (VA) curto e (4) resposta ventricular-atrial-ventricular (V-A-V) à estimulação ventricular overdrive (VOD) com intervalo pós-estimulação corrigido menos duração do ciclo de taquicardia (cPPI-TCL) > 110 EM. Demonstrando que tanto a via lenta (SP) quanto a via rápida (FP) são capazes de conduzir mais rápido que a duração do ciclo de taquicardia (TCL) durante o arrastamento (5). Durante o arrastamento auricular através do SP, o PPI-TCL do septo auricular é inferior a 50 ms (6). O avanço ou término da taquicardia via bloqueio no PS foi demonstrado pelo estímulo extra atrial (EEA) (7). Essas manobras também servem para excluir vias nodoventriculares ou nodofasciculares ocultas. Nos pacientes cuja fisiologia de via dupla pôde ser demonstrada por estímulo extra programado atrial (PES) ou cuja taquicardia não foi induzida, o salto retrógrado foi demonstrado com o PES do ventrículo direito. A infusão de isoproterenol foi administrada em pacientes nos quais a taquicardia não pôde ser induzida.

O sucesso do procedimento foi definido como a incapacidade de induzir taquicardia com PES ou a presença de apenas um único salto ou salto/eco pelo menos 20 minutos após a ablação. Se a taquicardia fosse induzida ou fosse observado salto/eco bilateral (círculo completo), a ablação era continuada.

A ablação por RF foi realizada com um cateter sem irrigação de 4 mm (7 FR RF Marinr, Medtronic, Minneapolis, EUA) aplicando energia entre 35-50 W a uma temperatura máxima de 50-55°C na região onde os sinais apropriados foram vistos da região anterior, região da óstio-anel tricúspide do seio coronário eletroanatomicamente. Nos casos em que foi observado ritmo nodal lento, a ablação foi continuada por 45-60 segundos e após a aplicação, a excitabilidade da taquicardia e a presença de via lenta foram verificadas com PES do seio coronário. Nos casos em que nenhum efeito foi alcançado, a ablação foi continuada até a região óstio-teto do seio coronário. A energia não foi aplicada acima do nível do teto do seio coronário. A crioablação foi realizada com criocateter de 6 mm (Freezor Xtra 6 mm, Medtronic, Minneapolis, EUA). Se não houvesse prolongamento do tempo PR ou bloqueio AV, a ablação era continuada por 4 minutos a -80°C.

Após o procedimento, os antiarrítmicos foram suspensos e todos os pacientes foram examinados no ambulatório após 3 meses ou antes caso apresentassem sintomas sugestivos de recorrência. Quando foram encontrados eletrocardiograma ou sintomas sugestivos de recidiva, foi recomendado uso adicional de antiarrítmico ou repetição do procedimento.

O desfecho primário em nosso estudo foi definido como o sucesso alcançado imediatamente após o procedimento com ambos os métodos. A recorrência foi definida como a documentação de TRNAV (eletrocardiograma-ECG recente) ou TRNAV induzível. Os desfechos secundários foram os tempos de procedimento, os tempos de fluoroscopia e o aparecimento de complicações. Além disso, um acompanhamento de um ano ou mais foi realizado para todos os pacientes para avaliar qualquer ocorrência de TRNAV.

Após o procedimento, os pacientes foram convidados para um controle policlínico de rotina a cada 3 meses por até 2 anos. O acompanhamento rotineiro do ECG Holter não foi realizado porque não houve palpitação ou qualquer outra queixa nos demais pacientes, exceto em 3 pacientes do grupo crioablação que tiveram recorrência. Nenhum paciente foi perdido no acompanhamento e nenhuma mudança no tratamento médico foi feita.

### Análise estatística

A análise estatística foi realizada no programa SPSS 25.0 (IBM Corporation, Armonk, Nova York, Estados Unidos). As variáveis contínuas foram expressas como média (±desvio padrão) ou mediana (percentis 25-75) de acordo com a normalidade dos dados, enquanto as variáveis categóricas foram descritas por meio de frequências absolutas (n) e relativas (%). A distribuição normal das variáveis foi avaliada pelo teste Shapiro-Wilk Francia, enquanto a homogeneidade da variância foi avaliada pelo teste Levene. Testes paramétricos (teste t para amostras independentes) ou não paramétricos (teste U de Mann-Whitney) foram utilizados com os resultados de Bootstrap para variáveis contínuas, enquanto as variáveis categóricas foram analisadas através dos testes Qui-Quadrado de Pearson e Exato de Fisher com o método de Monte Carlo. Além das variáveis independentes, as variáveis dependentes para comparação intragrupo estão apresentadas na tabela. O teste de McNemar foi utilizado no caso de comparações intragrupos, que trata de amostras dependentes. A técnica de simulação e as relações das colunas foram comparadas entre si e demonstradas de acordo com os resultados do valor p corrigido de Benjamini-Hochberg. Um valor de p inferior a 0,05 foi considerado significativo. O nível de significância adotado na análise estatística foi de 5%.

## Resultados

As características demográficas e processuais dos pacientes submetidos à ablação estão relatadas nas Tabelas 1 e 2. Setenta e três pacientes com diagnóstico de TRNAV foram incluídos no estudo. Trinta e dois foram submetidos à crioablação e 41 à ablação por RF. De todos os pacientes incluídos no estudo, quarenta e oito (65,8%) eram do sexo feminino e 25 (34,2%) do sexo masculino. A média de idade dos pacientes submetidos à crioablação e à ablação por RF foi de 47,2±14,9 anos e 48,8±15,0 anos, respectivamente (p=0,62). Após o tratamento ablativo, os pacientes submetidos à crioablação e os pacientes submetidos à ablação por RF foram acompanhados por uma mediana de 16 meses (p=0,542). Não houve diferença estatisticamente significativa entre os dois grupos em termos dos tempos médios de duração do ciclo basal (BCL) e duração do ciclo de taquicardia (TCL) (p=0,618, p=0,854, respectivamente) (Tabela 1; Figura Central).

**Tabela 1:** – Características demográficas e de procedimento dos pacientes submetidos à ablação

	Crioablação (n=32)	Ablação por RF (n=41)	Valor p
**Gênero, n (%)**			0,214ᶜ
Fem.	24 (75,0)	24 (58,5)	
Masc.	8 (25,0)	17 (41,5)	
**Idade (anos), média (±DP)**	47,16 (±14,94)	48,83 (±15,05)	0,623ᵗ
**Sucesso, n (%)**			0,068ᶠ
Sim	29 (90,6)	41 (100,0)	
Não	3 (9,4)	0 (0,0)	
**Tempo de procedimento (min), mediana (q1/q3)**	79,5 (64,5/97)	68 (54/89)	0,129ᶸ
**Tempo de fluorescência (min), mediana (q1/q3)**	7,55 (5,6/11,55)	17 (9,5/25)	<0,001ᶸ
**Acompanhamento (mês), mediana (q1/q3)**	18 (16/20,5)	16 (14/19)	0,542
**BCL (ms**), **média (DP)**	781,22 (136,03)	798,29 (145,58)	0,618u
**AH (ms**), **mediana (q1/q3)**	98 (80/101)	80 (74/82)	0,004ᶸ
**HV (ms), mediana (q1/q3)**	42 (40/44)	39 (34/42)	0,015ᶸ
**TCL (ms), mediana (q1/q3)**	325 (295/375)	330 (300/386)	0,854ᶸ

ᶜTeste Qui-Quadrado de Pearson, ᶠTeste exato de Fisher, ᵘTeste Mann Whitney u, ᵗTeste T de amostras independentes (Bootstrap), ou Razão de chances (intervalo de confiança de 95%), q1: percentil 25, q3: percentil 75, DP: desvio padrão; BCL: comprimento do ciclo basal; AH: átrio-his; HV: his-ventricular; TCL: comprimento do ciclo de taquicardia.

Bloqueio AV temporário foi observado em três pacientes em ambos os grupos (p>0,05), enquanto nenhum paciente apresentou bloqueio AV total permanente durante e após o procedimento. Não houve diferenças significativas quanto ao sucesso do procedimento (p= 0,068). Nenhum salto ou eco único foi detectado em 30 (41,1%) pacientes em todos os pacientes submetidos à ablação. No acompanhamento pós-procedimento, foi detectada recidiva de taquicardia em 3 pacientes do grupo crio, enquanto nenhuma recidiva foi encontrada no grupo RF (p=0,057). O período refratário efetivo do nó atrioventricular (PRENAV) pré-procedimento foi 230ms e 245 ms nos grupos de crioablação e ablação por RF, enquanto nenhuma alteração estatisticamente significativa foi detectada nos tempos PRENAV pós-procedimento. Por outro lado, o prolongamento dos tempos PRENAV dentro dos intragrupos foi estatisticamente significativo (p<0,001). Os tempos de Wenckebach anteriores antes e após o procedimento foram semelhantes nos grupos crioablação e ablação por RF (p>0,05). O prolongamento dos tempos anteriores de Wenckebach dentro dos intragrupos foi, entretanto, estatisticamente significativo (p<0,001) (Tabela 2).

**Tabela 2:** – Características processuais de pacientes submetidos à ablação

	Crioablação (n=32)	Ablação por RF (n=41)	valor p
**Salto pós-procedimento (sem eco), n (%)**			0,244ᶜ
Sim	7 (21,9)	8 (19,5)	
Não	25 (78,1)	33 (80,5)	
**Eco pós-procedimento (sem salto),n (%)**			0,038ᶜ
Sim	1 (3.2)	4 (9,8)	
Não	31 (96,8)	37 (90,2)	
**Salto e eco pós-procedimento, n (%)**			0,168ᶜ
Sim	11 (34,4)	12 (29,3)	
Não	21 (65,6)	29 (70,7)	
**Bloqueio AV temporário, n (%)**			0,999ᶠ
Sim	3 (9,4)	3 (7,3)	
Não	29 (90,6)	38 (92,7)	
**Recorrência de taquicardia pós-procedimento, n (%)**			p=0,057^f^
Sim	3 (9,4)	0 (0,0)	
Não	29 (90,6)	41 (100,0)	
**PRENAV (ms), mediana (q1/q3)**			
Pré-Procedimento	230 (210/240)*	245 (230/275)**	0,020ᶸ
Pós-Procedimento	290 (270/310)*	280 (250/320)**	0,580ᶸ
Mudança (PréP-PósP)	60 (40/70)	40 (-5/65)	0,011ᶸ
**Wenckebach anterior (ms), mediana (q1/q3)**			
Pré-Procedimento	300 (300/300)***	300 (300/310)****	0,259ᶸ
Pós-Procedimento	340 (315/350)***	340 (320/380)****	0,231ᶸ
Mudança (PréP-PósP)	30 (10/40)	35 (10/70)	0,464ᶸ

*p<0,001 **p<0,001 ***p<0,001 ****p<0,001 (valor de p para intragrupo) ᶜTeste qui-quadrado de Pearson, ᶠTeste exato de Fisher, ᵘTeste Mann Whitney u ou Odds Ratio (intervalo de confiança de 95%), q1: percentil 25, q3: percentil 75, DP: desvio padrão; AV: átrio-ventricular; PRENAV: período refratário efetivo do nó atrioventricular.

## Discussão

As opções de tratamento para pacientes com TRNAV sintomática consistem em tratamento médico, com alguns antiarrítmicos de classes I, II e IV, e ablação. Contudo, a maioria dos pacientes com TRNAV são relativamente saudáveis e jovens, e a ablação ganhou destaque, em vez da terapia medicamentosa de longo prazo.^8^ A ablação por cateter é uma importante opção de tratamento para pacientes sintomáticos, pois melhora significativamente a qualidade de vida e reduz custos. As indicações atuais para ablação incluem sintomas persistentes resistentes à terapia médica e à preferência do paciente. Contudo, as recomendações variam de acordo com a categoria da TSV e as taxas de sucesso.^9^

Diferentemente de alguns estudos,^6^ que relataram maior tempo de procedimento com a crioablação, nosso estudo mostra que, em pacientes tratados com crioablação, o tempo de fluoroscopia foi significativamente menor (7,55 [5,6-11,55] min vs. 17 [9,5-25] min p<0,001). Esse resultado está de acordo com dados relatados em outros estudos.^10^ Mesmo que o sucesso agudo do procedimento não seja diferente entre os dois métodos, a crioablação pode ser considerada vantajosa, em comparação ao método RF, pois pode ser realizada com segurança com criomapeamento durante taquicardia, a linha de demarcação é feita com uma área de ablação focal mais clara, cria uma lesão trombogênica baixa e proporciona uma posição mais estável do cateter.^4,6^ Ao final do acompanhamento do nosso estudo, a recorrência tardia da taquicardia foi observada em 3 pacientes tratados com crioablação, enquanto nenhuma recorrência foi encontrada com a ablação por RF, não mostrando diferença entre os dois métodos (p=0,057). Nosso resultado está de acordo com a prevalência de recorrência relatada por Kimman et al.^11^ que demonstraram taxa de recorrência semelhante (9% e 10% em pacientes tratados com RF e crioablação, respectivamente) e com os resultados obtidos por Chaumont et al.^12^

Devido ao risco muito baixo de bloqueio AV durante a crioablação, tornou-se possível tratar a TRNAV durante a arritmia. Além disso, embora um bloqueio AV temporário seja algumas vezes observado durante a crioablação, um bloqueio AV permanente é extremamente raro,^13,14^ diferentemente da ablação por RF.^15^ Mesmo em centros experientes, a incidência de bloqueio AV completo permanente que requer implante de marca-passo, foi relatada em aproximadamente 1%.^8,16^ O risco de bloqueio AV completo com ablação por RF continua sendo um problema significativo. Na literatura, as taxas de recorrência de taquicardia têm sido relatadas como bastante baixas, independentemente dos métodos de ablação.^17^ Porém, no acompanhamento dos pacientes submetidos à crioablação, não houve necessidade de uso de antiarrítmicos e repetição do procedimento.

Além disso, situações em que o alvo da ablação está próximo do nó AV, como em TRNAV, em pacientes jovens com triângulos de Koch menores tornam a crioablação uma opção mais segura.^18^

### Limitações

Os resultados do nosso estudo devem ser considerados preliminares. Embora os grupos do nosso estudo tenham sido semelhantes em termos de idade e tipos de TRNAV, as principais limitações do nosso estudo são a retrospectiva e o pequeno número de pacientes. O pequeno número de pacientes pode não ter criado diferença entre os grupos em termos de recorrência de arritmia. Também não houve abordagem uniforme em relação à entrega de lesões de segurança adicionais após uma lesão de crioablação inicialmente bem-sucedida.

## Conclusão

Os resultados do nosso estudo mostram que a crioablação requer menos tempo de fluoroscopia e é uma alternativa segura e não inferior à ablação por RF em pacientes com TRNAV. Tanto a crioablação quanto a RF têm taxas de sucesso comparáveis e satisfatórias na ablação de TRNAV. Historicamente, a crioablação parece ter uma taxa de recorrência ligeiramente mais elevada durante o acompanhamento a longo prazo: para melhorar estes resultados, é fundamental respeitar os parâmetros de ablação firmes. O risco de criação de bloqueio AV inadvertido continua a ser um problema importante no uso de energia de RF, tornando-a menos adequada para uso em pacientes jovens e fisicamente ativos.
