# Supplementary material for: Ablação de Taquicardia Reentrante Nodal Atrioventricular com Crioablação Focal, Comparada com Ablação por Radiofrequência: Experiência em Centro Único
Source: Arq Bras Cardiol. 2024 Sep 17;121(9):e20230604. [Article in Portuguese] doi: 10.36660/abc.20230604 (PMC11495587; doi:10.36660/abc.20230604)
Supplement: Translation

# Translation: Ablation of Atrioventricular Nodal Reentrant Tachycardia with Focal Cryoablation, Compared with Radiofrequency Ablation: Single-Center Experience

**DOI:** 10.36660/abc.20230604i

**Authors:** Caner Topaloğlu, Francesco Fici, Philippe van de Borne, Uğur Taşkin, Mustafa Dogdus, Serkan Saygi, Istemihan Tengiz

**Affiliations:** 1 Turkey Izmir University of Economics - Department of Cardiology, Izmir – Turkey; 2 Spain University of Salamanca - Department of Cardiovascular Risk, Salamanca – Spain; 3 Italy University of Milan-Bicocca – Cardiology, Milano – Italy; 4 Belgium Université Libre de Bruxelles - Department of Cardiology, Bruxelles – Belgium; 5 Belgium Erasmus Hospital - Department of Cardiology, Bruxelles – Belgium; 6 Sweden Karolinska University Hospital – Cardiology, Stockholm – Sweden

**Keywords:** Atrioventricular Nodal Reentry Tachycardia, Cryosurgery, Radiofrequency Ablation

## Abstract

**Background:**

The ablation of atrioventricular nodal reentrant tachycardia (AVNRT) with cryoablation is an alternative to radiofrequency (RF) ablation in patients due to the low risk of total atrioventricular block. An increase in early-late recurrences after cryoablation is reported as an important disadvantage.

**Objectives:**

In this study, we aimed to compare the acute procedural success and the long-term recurrence rates of patients, with AVNRT who underwent methods.

**Methods:**

73 patients with AVNRT were included in the study: 32 with cryoablation and 41 with RF ablation. There was no statistically significant difference between acute procedural success in methods. The ablation procedure was performed by an operator experienced in arrhythmology. The choice of RF or cryoablation was made in the electrophysiology laboratory based on the material already available during the procedure. After the procedure, the patients were evaluated every 3 months for 2 years in polyclinic control. The significance level adopted in the statistical analysis was 5%.

**Results:**

The 2 groups of patients were homogeneous. The fluoroscopy time (p<0.001) was shorter, but atrium-his (p=0.004) and his-ventricular (p=0.015) times were longer in the cryoablation group. There was no significant difference, in terms of acute procedural success, post-procedure jump without a single echo, and presence of echo and jump.

**Conclusions:**

Cryoablation requires less fluoroscopy time and is a safe non-inferior alternative to RF ablation in patients with AVNRT. The risk of AV block is a significant problem with the use of RF energy, making it less suitable for use in young and physically active patients.

## Introduction

Supraventricular tachycardia (SVT) is a general term to describe a group of arrhythmias, whose mechanism includes, or is above, the atrioventricular node. Atrioventricular nodal re-entrant tachycardia (AVNRT) is the most common SVT in adults, with an overall prevalence of 0.2%.^1-3^ The mechanisms of the vast majority of SVT forms have been elucidated and significant developments have been achieved in pharmacological and interventional treatments.^4^

Generally, guidelines^5^ recommend ablation for patients with recurrent or poorly tolerated SVT despite pharmacological treatment. When current guidelines recommendations are considered, catheter ablation is the preferred treatment modality for most patients, regardless of the presence of structural heart disease. Currently, there are two options for slow pathway ablation: focal cryoablation and radiofrequency (RF) ablation. Both techniques have different advantages and disadvantages.^6^

Focal cryoablation is used as an alternative to RF ablation in selected patients due to the absence of permanent atrioventricular (AV) block risk. However, an increase in early and late recurrences after cryoablation, compared to RF ablation is reported as the most important disadvantage of this method.^7^

## Objective

Our retrospective study aimed to compare cryoablation with RF ablation in terms of procedural success rate and the long-term follow-up results of patients with AVNRT.

## Methods

The current retrospective study was performed in patients who underwent AV node slow pathway ablation for AVNRT. Seventy-three patients, selected from medical records of the catheter laboratory database, were included in the study. The entire study population consisted of patients (73 patients) who underwent ablation with the diagnosis of AVNRT in the 6 months between January 2020 and June 2020. No patients were excluded from the analysis. The ablation procedure was performed by an operator who is highly experienced in the field of arrhythmology. The choice of RF or cryoablation was made in the electrophysiology laboratory based on the material already available during the procedure. There was no other bias in terms of technique choice. The operator routinely performed the ablation procedures every weekday afternoon. The cost of the materials used in both techniques was not burdensome for society and/or the patient and did not affect the chosen technique. The study was conducted following the Declaration of Helsinki and was approved by a local independent Ethics Committee.

AVNRT was diagnosed using criteria as follows according to previous reports: (1) evidence of dual AV nodal physiology, (2) tachycardia initiation by atrial drive train with Atrial-His-Atrial (A‐H‐A) response, (3) short septal ventricle-atrial (VA) time, and (4) ventricular‐atrial‐ventricular (V‐A‐V) response to ventricular overdrive (VOD) pacing with corrected post pacing interval minus tachycardia cycle length (cPPI‐TCL) > 110 ms. Demonstrating that both the slow pathway (SP) and fast pathway (FP) are capable of conducting faster than the tachycardia cycle length (TCL) during entrainment (5). During atrial entrainment via the SP, atrial septal PPI‐TCL is less than 50 ms (6). Advancement or termination of the tachycardia via block in the SP was demonstrated by atrial extra stimulus (AES) (7). These maneuvers also serve to exclude concealed nodo-ventricular or nodo-fascicular pathways. In patients, whose dual pathway physiology could be demonstrated by atrial programmed extra stimulus (PES) or whose tachycardia was not induced, the retrograde jump was demonstrated with the PES from the right ventricle. Isoproterenol infusion was administered in patients in whom tachycardia could not be induced.

Procedural success was defined as the inability to induce tachycardia with PES or the presence of only a single jump or jump/echo at least 20 minutes after ablation. If tachycardia was induced or bilateral jump/echo (full circle) was observed, ablation was continued.

RF ablation was performed with a 4 mm non-irrigation catheter (7 FR RF Marinr, Medtronic, Minneapolis, USA) by applying energy between 35-50 W at max 50-55°C to the region where appropriate signals were seen from the anterior region of the coronary sinus ostium-tricuspid annulus region electro-anatomically. In cases where a slow nodal rhythm was observed, ablation was continued for 45-60 seconds and after the application, the excitability of tachycardia and the presence of a slow pathway were checked with coronary sinus PES. In cases where no effect was achieved, ablation was continued up to the coronary sinus ostium-roof region. The energy was not applied above the coronary sinus roof level. The cryoablation was performed with a 6 mm cryo catheter (6-mm Freezor Xtra, Medtronic, Minneapolis, USA). If there were no PR time prolongation or AV block, the ablation was continued for 4 minutes at -80°C.

After the procedure, antiarrhythmic drugs were discontinued and all patients were checked in the outpatient clinic after 3 months or earlier if they had symptoms suggestive of recurrence. When an electrocardiogram or symptoms suggestive of recurrence were found, additional antiarrhythmic use or a repeat procedure was recommended.

The primary endpoint in our study was defined as the success achieved immediately after the procedure with both methods. Recurrence was defined as the documentation of AVNRT (recent electrocardiogram-ECG) or inducible AVNRT. Secondary endpoints were the procedure times, the fluoroscopy times, and the appearance of complications. Moreover, a follow-up of one year or more was performed for all patients to assess any AVNRT occurrence.

After the procedure, the patients were invited to a routine polyclinic control every 3 months for up to 2 years. Routine ECG Holter follow-up was not performed because there was no palpitation or any other complaint in the other patients, except for 3 patients in the cryoablation group who had recurrence. In the follow-up, no patient was lost and no change in medical treatment was made.

### Statistical analysis

Statistical analysis was performed with the SPSS 25.0 (IBM Corporation, Armonk, New York, United States) program. Continuous variables were expressed as mean (±standard deviation) or median (25th-75th percentiles) according to data normality, while categorical variables were described using absolute (n) and relative (%) frequencies. The normal distribution of the variables was evaluated by the Shapiro-Wilk Francia test, while the homogeneity of variance was evaluated by the Levene test. Parametric tests (independent samples t-test) or non-parametric (Mann-Whitney U test) were used with the Bootstrap results for continuous variables, while categorical variables were analyzed using the Pearson Chi-Square and Fisher Exact tests with the Monte Carlo method. In addition to the independent variables, the dependent variables for intra-group comparison are stated in the table. McNemar test was used in the case of intragroup comparisons, which deals with dependent samples. The simulation technique and column ratios were compared with each other and demonstrated according to Benjamini-Hochberg’s corrected p-value results. A p-value of less than 0.05 was considered significant. The significance level adopted in the statistical analysis was 5%.

## Results

Demographic and procedural characteristics of patients undergoing ablation are reported in Tables 1 and 2. Seventy-three patients with a diagnosis of AVNRT were included in the study. Thirty-two underwent cryoablation and 41 RF ablation. Of all the patients included in the study, forty-eight (65.8%) were female and 25 (34.2%) were male. The mean age of the patients who underwent cryoablation and RF ablation were 47.2±14.9 years and 48.8±15.0 years, respectively (p=0.62). After the ablation treatment patients who underwent cryoablation and patients who underwent RF ablation were followed up for a median of 16 months (p=0.542). There was no statistically significant difference between the two groups in terms of mean basal cycle length (BCL) and tachycardia cycle length (TCL) times (p=0.618, p=0.854, respectively) ( Table 1; Central Illustration).

**Table 1:** – Demographic and procedural characteristics of patients undergoing ablation

	Cryoablation (n=32)	RF-Ablation (n=41)	p-value
**Gender, n (%)**			0.214ᶜ
Female	24 (75.0)	24 (58.5)	
Male	8 (25.0)	17 (41.5)	
**Age (years), mean (±SD)**	47.16 (±14.94)	48.83 (±15.05)	0.623ᵗ
**Success, n (%)**			0.068ᶠ
Yes	29 (90.6)	41 (100.0)	
No	3 (9.4)	0 (0.0)	
**Procedure time (min), median (q1/q3)**	79.5 (64.5/97)	68 (54/89)	0.129ᶸ
**Fluoro-time (min), median (q1/q3)**	7.55 (5.6/11.55)	17 (9.5/25)	<0.001ᶸ
**Follow-up (month), median (q1/q3)**	18 (16/20.5)	16 (14/19)	0.542
**BCL (ms), mean (SD)**	781.22 (136.03)	798.29 (145.58)	0.618u
**AH (ms), median (q1/q3)**	98 (80/101)	80 (74/82)	0.004ᶸ
**HV (ms), median (q1/q3)**	42 (40/44)	39 (34/42)	0.015ᶸ
**TCL (ms),median (q1/q3)**	325 (295/375)	330 (300/386)	0.854ᶸ

ᶜPearson Chi-Square test, ᶠFisher Exact Test, ᵘMann Whitney u test, ᵗIndependent Samples T-test (Bootstrap), orOdds Ratio (95% Confidence interval), q1: percentile 25, q3: percentile 75, SD: standard deviation; BCL: basal cycle length; AH: atrium-his; HV: his-ventricular; TCL: tachycardia cycle Length.

Temporary AV block was observed in 3 patients in both groups (p>0.05), while no patient had a permanent total AV block during and after the procedure. There were no significant differences regarding procedural success (p= 0.068). No jump or single echo was detected in 30 (41.1%) patients in all patients who underwent ablation. In the post-procedure follow-up, tachycardia recurrence was detected in 3 patients in the cryo-group, while no recurrence was found in the RF group (p=0.057). Atrioventricular node effective refractory period (AVNERP) time pre-procedure was 230 ms and 245 ms in the cryoablation and the RF ablation groups, while no statistically significant change was detected in postprocedure AVNERP times. Conversely, the prolongation of AVNERP times within the intragroups was statistically significant (p<0.001). Anterior Wenckebach times before and after the procedure were similar in the cryoablation and RF ablation groups (p>0.05). The prolongation of anterior Wenckebach times within the intragroups was however statistically significant (p<0.001) (Table 2).

**Table 2:** – Procedural characteristics of patients undergoing ablation

	Cryoablation (n=32)	RF-Ablation (n=41)	p-value
**Post-procedure jump (without eco), n (%)**			0.244ᶜ
Yes	7 (21.9)	8 (19.5)	
No	25 (78.1)	33 (80.5)	
**Post-procedure echo (without jump), n (%)**			0.038ᶜ
Yes	1 (3.2)	4 (9.8)	
No	31 (96.8)	37 (90.2)	
**Post-procedure jump and echo, n (%)**			0.168ᶜ
Yes	11 (34.4)	12 (29.3)	
No	21 (65.6)	29 (70.7)	
**Temporary AV Block, n (%)**			0.999ᶠ
Yes	3 (9.4)	3 (7.3)	
No	29 (90.6)	38 (92.7)	
**Post-procedure tachycardia recurrence, n (%)**			p=0.057^f^
Yes	3 (9.4)	0 (0.0)	
No	29 (90.6)	41 (100.0)	
**AVNERP (ms), median (q1/q3)**			
Pre-Procedure	230 (210/240)*	245 (230/275)**	0.020ᶸ
Post-Procedure	290 (270/310)*	280 (250/320)**	0.580ᶸ
Change (PreP-PostP)	60 (40/70)	40 (-5/65)	0.011ᶸ
**Anterior Wenckebach (ms), median (q1/q3)**			
Pre-Procedure	300 (300/300)***	300 (300/310)****	0.259ᶸ
Post-Procedure	340 (315/350)***	340 (320/380)****	0.231ᶸ
Change (PreP-PostP)	30 (10/40)	35 (10/70)	0.464ᶸ

*p<0.001 **p<0.001 ***p<0.001 ****p<0.001 (p-value for intragroup). ᶜPearson Chi-Square test, ᶠFisher exact Test, ᵘMann Whitney u test, orOdds Ratio (95% Confidence interval), q1: percentile 25, q3: percentile 75, SD: standard deviation; AV: Atrio-Ventricular; AVNERP: Atrioventricular Node Effective Refractory Period.

## Discussion

Treatment options for patients with symptomatic AVNRT consist of medical treatment, with some class I, II, and IV antiarrhythmic drugs, and ablation. However, the majority of patients with AVNRT are relatively healthy and young, and ablation has come to the fore, instead of long-term drug therapy.^8^ Catheter ablation is an important treatment option for symptomatic patients, as it significantly improves the quality of life and reduces costs. Current indications for ablation include persistent symptoms resistant to medical therapy and patient preference. However, recommendations vary according to the SVT category and the success rates.^9^

Differently from some studies,^6^ that have reported a longer procedure time with the cryoablation, our study shows that, in patients treated with cryoablation, the fluoroscopy time was significantly lower (7.55 [5.6-11.55] min vs 17 [9.5-25] min. p<0.001). This result is in agreement with data reported in other studies.^10^ Even if the acute procedural success is not different between both methods, the cryoablation can be considered advantageous, compared to the RF method, because it can be safely performed with cryo-mapping during tachycardia, the demarcation line is made with a clearer focal ablation area, it creates a low thrombogenic lesion, and provides a more stable catheter position.^4,6^ At the end of the follow-up of our study, the late tachycardia recurrence was observed in 3 patients treated with cryoablation, while no recurrence was found with the RF ablation, showing no difference between the two methods (p=0.057). Our result is in agreement with the prevalence of recurrence reported by Kimman et al.^11^ which have shown a similar rate of recurrences (9% and 10% in patients treated with the RF and cryoablation, respectively) and with the results obtained by Chaumont et al.^12^

Because of the very low risk of AV block during cryoablation, it has become possible to treat AVNRT during arrhythmia. Furthermore, although a temporary AV block is sometimes observed during cryoablation, a permanent AV block is extremely rare,^13,14^ differently from the RF ablation.^15^ Even in experienced centers, the incidence of permanent complete AV block requiring pacemaker implantation has been reported to be approximately 1%.^8,16^ The risk of complete AV block with RF ablation remains a significant problem. In the literature, tachycardia recurrence rates have been reported to be quite low regardless of ablation methods.^17^ However, in the follow-up of the patients who underwent cryoablation, there was no need for anti-arrhythmic drug use and repetition of the procedure.

Moreover, situations where the ablation target is close to the AV node, as in AVNRT, in young patients with smaller Koch triangles make cryoablation a safer option.^18^

### Limitations

The results of our study must be considered as preliminary. Although the groups in our study were similar in terms of age and AVNRT types, the main limitations of our study are its retrospective and the small number of patients. The small number of patients may not have created a difference between the groups in terms of arrhythmia recurrence. There was also no uniform approach regarding the delivery of additional safety lesions after an initially successful cryoablation lesion.

## Conclusion

The results of our study show that cryoablation requires less fluoroscopy time and is a safe non-inferior alternative to RF ablation in patients with AVNRT. Both cryo- and RF ablation have comparable and satisfying success rates in AVNRT ablation. Historically cryoablation seems to have a slightly higher recurrence rate during long-term follow-up: to improve these results, it is paramount to respect firm ablation endpoints. The risk of creating inadvertent AV block remains a major issue in using RF energy, thus making it less suitable to be used in young and physically active patients.
